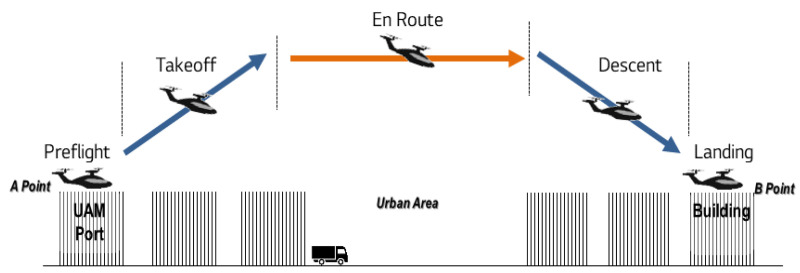# Urban Air Mobility Noise: Further Considerations on Indoor Space

**DOI:** 10.3390/ijerph191811298

**Published:** 2022-09-08

**Authors:** JungHoon Kim

**Affiliations:** Korea Institute of Civil Engineering and Building Technology, Goyang 10223, Korea; kimjunghoon@kict.re.kr

**Keywords:** urban air mobility noise, electric vertical take-off noise, landing noise, noise standard

## Abstract

Various countries are preparing for the introduction of urban air mobility (UAM) vehicles, which move freely within the space above a city, as a new means of urban transportation. However, UAM vehicles present new forms of challenges to many urban residents. This study aims to propose newly sensory standards for the noise levels of UAM vehicles in urban indoor spaces based on two fundamental questions: (1) Would UAM vehicles not have a lower and wider impact on city residents than a commercial aircraft? (2) Should the flight of UAM vehicles not consider the sensory noise, like the conventional noise standard? UAM vehicles, unlike commercial aircrafts, will cause noise pollution in a broad area of the city. Therefore, expanded aircraft noise standards will be required. In addition, the hybridized noise generated by conventional vehicles in the ground and UAM vehicles in the air will affect urban residents. Furthermore, urban residents will be exposed to sensory noise from UAM vehicles, which fly directly above them but not within their line of sight. Therefore, the noise standards for UAM vehicles should include the sensory properties in addition to the physical properties in the existing noise standards.

## 1. Introduction

Rapid urbanization is clarifying the limits of urban spaces [1,2]. A typical limitation is the lack of road space on the ground [3]. Roads are essential for logistics and the transportation of people. However, there is a limit to the physical expansion of roads in urban spaces [4]. Therefore, the utilization of the upper spaces of cities is being considered. A representative method of utilizing the upper spaces of cities is to use an existing flying vehicle, such as an aircraft or helicopter. However, the upper spaces of cities are filled with numerous elements that interfere with flights, such as buildings, apartments, traffic lights, street lights, and electrical facilities. Therefore, the development of a type of aircraft intended for use in upper spaces of cities is in progress. Urban air mobility (UAM) is a representative form that involves the transportation of passengers or cargo using electric vertical take-off and landing (eVTOL) flying vehicles [5]. Various countries are establishing a new air-traffic-system concept that will operate UAM along with existing means in ground spaces, such as subways and buses [6]. However, most reports on UAM indicate that the research concerns are related to the vehicle flight and UAM port configuration methods.

Urban residents are experiencing problems from vehicular noise in their ground spaces. In a future where UAM will become common, urban residents will also suffer from noise in the upper spaces of cities. In other words, a hybrid noise standard addressing ground and upper space noises will be required. The results of previous studies suggested that the noise damages caused by the aircraft and sensory factors, such as fear and fright, are considerable for urban residents living near airports [7,8]. As aircrafts cause noise damage only within the places wherein they are located, the importance of the noise standard has not been prioritized [9]. However, stricter noise standards will be required for employing UAM, which is expected to be operated indiscriminately in urban spaces. Currently, noise standards are specified only for loudness and pitch, which are sound attributes of physical nature; however, because UAM vehicles fly in upper spaces of cities, additional standards for sensory noise are required.

The main consideration for planned UAM flights within a city is the comfort of city residents. While commercial aircrafts ensure fast logistics and transportation of people, urban residents living near the spaces where aircrafts take off, land, or move are affected considerably by noise [10]. Additionally, aircraft noise generated in the air extends to the ground, thus causing damage within a large radius [11]. Therefore, aircraft noise is observed and managed according to well-defined standards. Typically, the noise level of the aircraft is managed according to the effective perceived noise in decibels (EPNdB)-20 (Stage 2) standard established in 1960, with the aim of reducing it to the EPNdB-30 (Stage 5) level by 2030. To meet the EPNdB-30 standard, aircraft manufacturers and airports are continuously developing noise-reduction technologies [12]. However, in the case of UAM, the noise standards are not clear owing to the lack of flight data within the city. As UAM vehicles fly over the upper spaces of cities that can be blocked by buildings, it is inappropriate to introduce and use the aircraft noise standards tailored to open airports. Therefore, this study aims to suggest the noise standards for employing UAM in the future.

UAM crash or emergency landing situations due to in-flight problems will likely occur in an urban space populated by urban residents [13]. Nevertheless, the UAM-related noise issue must be overcome, as the goal of an aircraft is to transport objects or people efficiently over the shortest distance. Utilizing the upper spaces of cities in the future by UAM, just as the current road connects all the residential and commercial spaces in the city, will increase connectivity in all parts of the city.

## 2. Literature Review

### 2.1. Aircraft and UAM Noise Standards and Studies

Commercial aircrafts generate loud noise during take-off and landing [14]. Therefore, noise standards are implemented around airports where the aircrafts take-off and land [15]. Furthermore, measures for the noise generation of aircrafts themselves are managed by the aircraft type. Aircraft noise standards are expressed in weighted, equivalent, continuous, perceived, noise levels (WECPNL), which is a unit calculated by assigning the average value of the highest noise level (in dB) generated during aircraft flight and the flight number weight per time period (in dB) that represents only the instantaneous magnitude of noise [16]. Table 1 summarizes the effects of noise levels based on WECPNL on a residential environment.

The Federal Aviation Administration (FAA) suggests noise standards and is continuously investigating noise measurements from 10,328 households around 20 airports in the United States. As the standards for noise vary in each country, this study intends to summarize the cases in South Korea. South Korea utilized the figures from the FAA to enact and enforce a law on noise and vibration around airports [17]. Zones have been designated according to the noise level with corresponding noise countermeasures, as summarized in Table 2.

Noise countermeasures and standards are applied specifically to areas around airports; however, these existing noise countermeasures are insufficient for aircraft routes as commercial aircraft take-off and landing routes are at low altitudes in the city. No noise countermeasures are needed when an aircraft flies several kilometers during a cruise flight. However, as UAM vehicles operate at an altitude of approximately 300 to 600 m, just above the locations where city residents live, standards for noise during take-off, cruise flight, and landing of UAM vehicles must be implemented.

### 2.2. Civiel Airplane, Helicopter, and UAM Classification and Noise Level

There are two main types of aircrafts: fixed- and rotary-wing. Civil aircrafts and helicopters are examples of fixed- and rotary-wing aircrafts, respectively. Current UAM vehicles may possess various forms; however, they are typically a type of rotary-wing aircraft [18]. The classification and noise of various aircraft types are summarized in Table 3.

Both fixed- and rotary-wing aircrafts have lower take-off noise than landing noise [19]. Higher noise was assumed to affect urban residents, as the engine output is increased during take-off; moreover, the output is maximized as the aircraft ascends to the operating altitude within a short time. Conversely, during landing, the aircraft continues to fly at a low altitude as it approaches the runway. Therefore, noise damage is greater during landing than during take-off [20]. Unfortunately, UAM is still at a suggested target noise level of the entire flight process, and the noise of the UAM is being studied only through simulations. In particular, there are limited studies on the types of noise that can be generated when a UAM vehicle operates within a city. Thus, it is essential to study the effects of noise generated during the flight of UAM vehicles on urban residents.

### 2.3. UAM Policies

The establishment of UAM policies in major countries is in the early stage [21]. The FAA and European Aviation Safety Agency (EASA), which traditionally have great influences on the designs of aircraft policies, provide guidelines on the certification and various regulations for UAM aircrafts [22]. UAM is a significant market for future aviation. Therefore, numerous countries are proposing policies to lead the UAM market.

The United States, one of the UAM market leaders, is conducting a project to provide policy support to private companies with technological superiority. Specifically, since the early 2000s, the National Aeronautics and Space Administration (NASA) has been conducting research on door-to-door personal air vehicle exploration and early-stage UAM aircraft [23]. In 2022, the FAA announced guidelines on how to build a UAM vertiport, the basic infrastructure of UAM, to preoccupy the UAM industry [24]. In particular, research funds are provided for aircraft development by private companies to encourage their participation in UAM, establishing the necessary certification support for UAM commercialization and operation of the aviation area system.

Meanwhile, China is a strong player in the drone industry, in which a drone is a small UAM-type aircraft, and it aims to be recognized in the UAM market by introducing drone technology to UAM [25]. The Ehang Autoflight of China is presenting various types of eVTOL aircrafts with the intention of opening the UAM Taxi market by introducing the world’s first autonomous flying drone taxi. The Civil Aviation Administration of China allows unmanned aerial service pilot operation in 13 cities.

Europe is also establishing measures to efficiently manage the airspace over which UAM vehicles will fly. Air Traffic Management is being developed following the launch of the “Single European Sky” in 2004 to manage airspace across Europe in an integrated manner. In addition, the EASA is proposing vertical take-off, landing, and airframe standards and preparing regulations related to pilot certification. The EASA plans to commercialize passenger transportation after approving the use of UAM for small cargo transportation, such as courier services. Regulations on UAM vertiport operations and pilot licenses are also being prepared.

South Korea selected UAM as the next-generation national project and announced the Korea–UAM Roadmap for the integrated management of the basic infrastructure of UAM. In addition, UAM Team Korea was established in 2020 to develop and demonstrate UAM in cooperation with the private sector. A policy has been recently enforced to demonstrate that flight routes are being pursued with the goal of flying 30 UAM vertiports and 300 UAM vehicles per city [26].

Major cities around the world are supporting the adoption of UAM policies. A specific policy direction for classifying and operating UAM according to the listed purpose is presented, and the construction of infrastructure for UAM is in progress. However, prepared policy proposals for the possible noise levels caused by UAM vehicles are insufficient. For automobiles, the installation of a silencer to reduce noise pollution and the introduction of minimum distances between urban residents and the road were studied. These studies are also vital as UAM is being further developed.

## 3. Key Questions and Analysis

The noise standards of UAM currently being proposed have nearly followed the existing noise standards of commercial aircrafts. Currently, standards for commercial aircrafts focus on noise during take-off and landing, not at operating altitude. However, UAM vehicles will fly in the upper spaces of residential buildings. This raises the question of whether current UAM noise standards are appropriate. To address this, two key questions were formulated and addressed. Specifically, Section **3.1****. Question 1 (Related** to Spaces) and Section **3.2****. Question 2 (Related to Noise Affecting** Urban Residents) are as follows.

### 3.1. Question 1 (Related to Spaces)

(1) As commercial aircrafts fly on a fixed air route, noise is limited to the vicinity of the air route. (2) However, UAM vehicles fly at low altitudes in urban areas and are likely to have constantly changing air routes. (3) **Therefore, would UAM vehicles not have a lower and wider impact on city residents than commercial aircrafts?**

(1) Referring to Section 2.2. Civiel Airplane, Helicopter, and UAM Classification and Noise Level, aircraft noise standards mainly consider the take-off and landing phases. An assessment of the noise levels according to real-time aircraft movements in the Korea airport noise portal system indicates that the aircraft generates noise in the range of 85–90 dB during take-off and landing (Table 4). In particular, in the take-off phase, the generated noise is in the range of 75–90 dB over a distance of 5 km from the runway; however, the effect on urban residents is negligible when the aircraft ascends to a certain altitude or during a cruise flight.

As the aircraft moves between Airport (District A) ↔ Airport (District B), as shown in Table 4, the route is constant. Therefore, considering the distribution of aircraft noise according to the period, the noise pattern is practically the same; only the direction of the noise changes depending on the direction of take-off and landing. The average annual and monthly noise distribution in South Korea was in the range of 73.0–74.4 WECPNL at Gimpo Airport (113,580 flights in 2020) and 79.0–81.0 WECPNL at Jeju Airport (138,256 flights in 2020). Table 5 summarizes the results.

**Figure 1:**
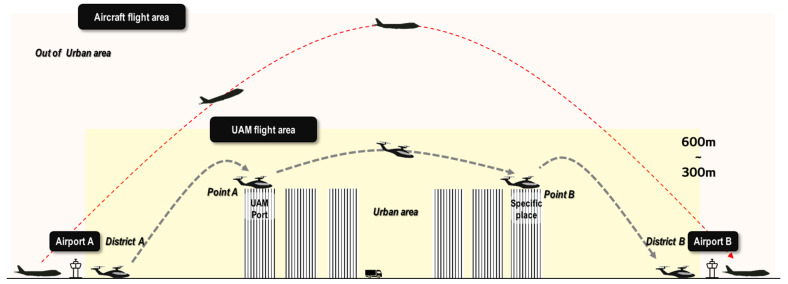
Flight routes of aircraft and UAM vehicles.

(2) As discussed in Section 2.3, take-off and landing are the main operational phases within city spaces, and the route is in the upper spaces just above the city. Meanwhile, the cruising altitude of UAM vehicles is in the range of 300 to 600 m, which is the same as the height at the time of the take-off and landing of an aircraft. This height is comparable to or lower than the height of a city building (Burj Khalifa: 828 m, Shanghai Tower: 632 m, and TAIPEI-101: 509 m). Thus, the flight height of UAM vehicles is comparable to the distance from a car in the ground space to the location of people inside the building. A simple comparison between the operations of a commercial aircraft and UAM vehicle is summarized in Figure 1.

A UAM aircraft can take-off and land at any point; therefore, it can freely fly on these routes: Airport A (District A) ↔ Airport B (District B), Airport A (District A) ↔ UAM Port (Point A), UAM Port (Point A) ↔ Specific place (Point B), and Specific place (Point B) ↔ Airport B (District B). Additionally, the route of each district and point will be different depending on the weather, wind, and aircraft conditions. Thus, predicting the flight path of the UAM aircraft will be a challenge.

(3) First, UAM vehicles fly at an altitude that is much lower than the flight path height of a commercial aircraft, as shown in Figure 1. This height is comparable to the height of buildings in the city. Second, the fluctuation range of the flight path is larger than that of commercial aircraft. Aircrafts fly on set routes; however, UAM vehicles change and move on various routes in real time. Therefore, the range of noise damage caused by UAM vehicles is lower than that of commercial aircrafts and will be applied more extensively.

### 3.2. Question 2 (Related to Noise Affecting Urban Residents)

**Figure 2:**
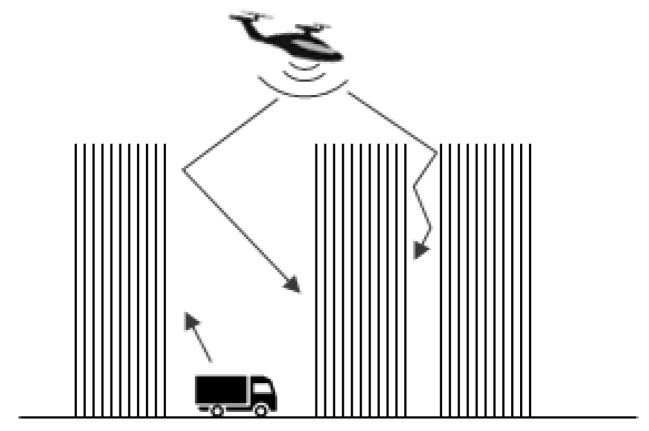
Prediction of noise distribution between UAM and automobiles. (Figure 2 is drawn by the author).

(1) Urban residents will be exposed to both the noise generated by existing vehicles in the ground and UAM vehicles in the air. (2) In particular, UAM vehicles will fly in the upper spaces of cities and outside the line of sight of urban residents. Noise from places outside the line of sight can cause discomfort and fear. (**3**) **Therefore, should the flight of UAM vehicles not consider the sensory noise, like the conventional noise standard?**

(1) Measures to reduce or block noise generated by UAM vehicles should be considered. However, noise isolation will be difficult for UAM. For example, while it is possible to reduce the noise in a specific section by installing a noise barrier around the automobile road, it is difficult to install a device to block the noise generated by UAM vehicles flying in the air shown in Figure 2.

The noise of a car spreads from lower to upper heights [27] and directly affects urban residents [28]. However, UAM vehicles flying over the city will cause extensive noise damage, owing to building reflections, and direct harm onto urban residents [29]. It is difficult to accurately present the range of noise damage patterns. Therefore, it is necessary to establish a standard for limiting noise and conduct basic research on hybrid noise between the ground and upper spaces.

(2) In addition to the noise associated with take-off and landing, aircrafts are affecting urban residents in various forms, such as loud noises during low-altitude flights [30], mental disorders caused by continuous exposure to aircraft noise [31], and sensory stimulation induced by aircraft noise [32]. In particular, helicopters generate extreme noise levels by flying at low altitudes, inflicting various types of discomfort directly on urban residents [33]. Among them, the flight characteristics of a helicopter are noteworthy in that it flies above the locations of urban residents, wherein visibility is blocked by buildings, thereby making it difficult to determine its exact location. The flight characteristics of a UAM vehicle are very similar to those of a helicopter [34].

Among the impacts caused by a helicopter flight are sensory discomforts, which should be considered as follows: the fear generated in situations in which urban residents cannot respond, owing to the sudden appearance of a helicopter from a place outside their line of sight or that only the sound of the rotor is being heard; mental fear of uncontrollable events, such as the possibility of the helicopter falling; and fear of a large object flying nearby. Regarding the effects of industrial noise on workers, damage caused by sensory noise types, such as intermittent and continuous sounds, in addition to the effects of physical noise (dB) in the workplace, has been suggested [35]. Thus, UAM vehicles, require more intensive research on sensory noise in addition to physical noise, thus considering the characteristics of helicopters and the damage caused by noise in industrial sites.

(3) To understand sensory and physical noises, it is necessary to understand sound. Noise is a type of sound wave. Sound is composed of three elements: loudness, pitch, and timbre. Sound types and their characteristics are outlined in Table 5.

The currently proposed noise standard for UAM is to use the index of EPNdB, which is a combination of loudness (dB) and pitch (Hz). UAM noise standards account for and regulate loudness and pitch. Meanwhile, there are no regulation standards for sensory properties related to timbre. Table 6 summarizes the elements required to be defined for all stages of UAM.

The definition of the time period during which the UAM vehicle can fly, time limit for the UAM vehicle to fly in place, and time limit rotor rotation before UAM flight should be considered to establish clear standards. Furthermore, among the types of timbre, continuous, interrupted, impulsive, background, fluctuating, and intermittent noise should be considered in detail (Table 7).

Table 8 summarizes the items necessary to expand the noise standard for each flight stage of UAM considering the continuous, interrupted, impulsive, background, fluctuating, and intermittent noise of timbre.

Noise is a subjective characteristic. Therefore, it is impossible to predict or reduce noise damage for all urban residents under the same basis. However, considering the characteristics of UAM vehicles that will fly directly above urban residents, it is clear that noise standards should be strengthened. In addition to the conventional physical noise standards, the materialization of the sensory noise standards is expected to lead to a system that resolves a significant part of the insensitivity to UAM in the city, as well as the development of UAM technologies.

## 4. Conclusions

Rapid urbanization has resulted in capacity restrictions of movements in ground spaces in cities. Therefore, UAM is being introduced to utilize the upper spaces of cities that have not been used yet. UAM has advantages regarding transportation, as it is free from traffic jams on roads. However, the flight of UAM vehicles within the city is expected to cause more complicated inconvenience than that of commercial aircrafts. Specifically, UAM vehicles will fly directly above urban residents, moving outside the line of sight, unlike cars in ground spaces. The noise generated by UAM vehicles will require new noise standards different from those for commercial aircrafts and automobiles. However, the majority of research on UAM has focused on the development of vehicles and configuration of the UAM port. Research on noise discomfort caused by UAM vehicles to urban residents is essential but insufficient.

In this study, two main questions were presented, and the amount of noise that is expected to be generated in employing UAM and the discomfort of noise affecting urban residents were addressed and summarized. Based on this process, newly sensory noise standards were proposed for UAM in urban indoor spaces. The results of this study are summarized as follows. First, UAM vehicles, unlike commercial aircrafts, will cause noise pollution in a broad area of the city. Therefore, expanded aircraft noise standards will be required. Second, urban residents will be affected by the hybridized noise generated by conventional vehicles in the ground and UAM vehicles in the air. Furthermore, urban residents will be exposed to sensory noise from UAM vehicles flying directly over their heads, which will not be visible but sensed intuitively. Therefore, the noise standards for UAM should include the sensory properties in addition to the physical properties in the existing noise standards.

In the future, UAM will become the main means of transportation within cities. Therefore, without applying more detailed noise standards suitable for UAM, urban residents will suffer from sensory noise, such as discomfort and fear, rather than experience convenience. The issue of point-to-point transportation noise will not be raised for transportation between buildings within the city where test flights are currently underway. However, with flying within the city applied in earnest, the current airplane noise standards cannot be applied equally to UAM vehicles. In other words, it is necessary to further study the noise generated during a point-to-point test flight in a city before UAM vehicles can fly in earnest.

More precise UAM noise standards should be set to allow the technological development of UAM. It is positive that the noise level of UAM is lowered considerably by the current airplane noise standards. However, in the vicinity of the UAM flight route and port, various damages are expected, owing to different types of noises compared with those inflicted by existing noise types. Therefore, more detailed noise standards, including sensory standards, should be introduced.

The limitations of this study are as follows. First, data on UAM could not be obtained. Therefore, the effect of UAM noise could not be presented accurately. Future UAM research must be conducted with newly generated UAM noise data. Second, only the classification on the sensory property of noise is currently presented, and studies on its effects are insufficient. Additionally, the effect of noise owing to sensory properties is subjective, which is applied differently to each individual. Therefore, in future research, objective standards and numerical values should be presented in conjunction with conducted research on the specific effects of sensory noise. Third, this paper focuses on reviewing the ongoing development of the UAM standards and existing indices. However, the WHO recognizes various noise indices as affecting human health, such as Ldn, Lden, Lnight, and LAmax [36]. Thus, future research may also consider other indices, particularly focusing on the effects on residents.

## Figures and Tables

**Table 1:** Effects of aircraft noise on a residential environment [16].

WECPNL	Loudness	Effects on a Residential Environment
*90*	very noisy	difficult to live in
*80–89*	noisy	requires the installation of soundproofing facilities for residential buildings
*76–79*	slightly noisy	requires the installation of soundproofing facilities for schools and hospitals
*71–75*	not noisy	sufficient for living
*≤70*	not noisy at all	comfortable for living

**Table 2:** Noise classification and countermeasures in the vicinity of Korean airports [17].

WECPNL	Zone		Division	Noise Countermeasures	
*≥95*	zone 1		noise-affected area	proposal of relocation measures installation of soundproofing facilities in schools and houses	land purchase and loss compensation
*90–95*	zone 2			support for the installation of soundproofing facilities in schools and houses; support for the installation of cooling facilities for schools and houses; support for school and home electricity bills; support for broadcast license fee	
*85–90*	zone 3	district A	Expected noise-affected area		not applicable
*80–85*		district B			
*75–80*		district C			

**Table 3:** Aircraft classification and noise levels (measured on runways).

Classification			Noise Level (EPNdB)		Remark
			Take-Off	Landing	
Fixed-wing	large size	Boeing 747–400B	96.8	101.8	average EPNdB, depending on measurement conditions
		Airbus 330–600R	92.1	101.7	
	middle size	Boeing 737–800	88.6	96.4	
Rotary-wing	helicopter	McDonnell Douglas 500	88.4	86.2	
		Eurocopter AS350	89.7	91.3	
	urban air mobility (UAM)	Bell Nexus (*5-seater*)	67 (target)		UAM is only presenting target figures
		City Airbus (*4-seater*)	70 (target)		
		Ehang 216	100 (target at 100 ft away)		
		Hyundai S-A1 (*5-seater*)	60 (target)		

**Table 4:** Real-time aircraft noise at Gimpo and Jeju Airports (as of 14 p.m., 25 August 2022 https://www.airportnoise.kr/anps/gis).

Gimpo Airport Aircraft Noise Level, Korea	Jeju Airport Aircraft Noise Level, Korea
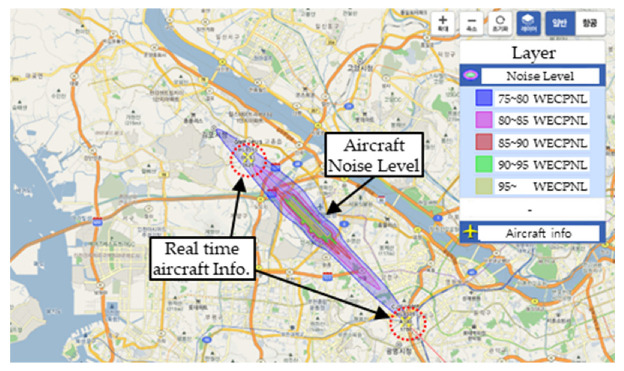	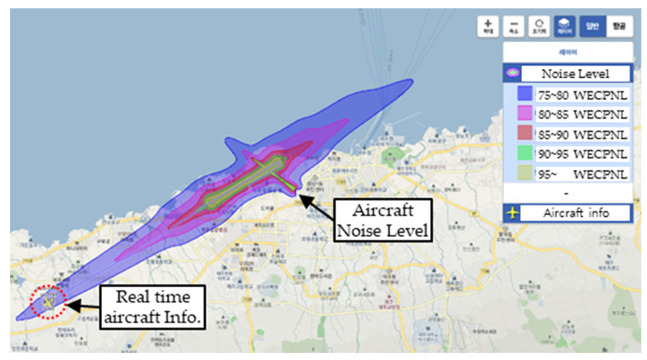

**Table 5:** Classification and characteristics of sounds.

Classification of Sounds	Description	Characteristic
loudness	loudness of the sound	physical properties
pitch	pitch of the sound related to the frequency of the sound	physical properties
timbre	sensory characteristics due to differences in sound components	sensory properties

**Table 6:** Sound regulation standard status and types of sounds.

Classification of Sounds	Regulation Standards	Type	
loudness	enact	decibel (dB), phon, sone	dimensionless unit used in electrical engineering, acoustic engineering, etc.
pitch	enact	Hertz (Hz), cent, cycle per second	number of waves per second
*timbre*	*not enacted*	*continuous noise*	has the same loudness throughout the day sound that repeats more than once per second, repeated interrupted noise sounds like continuous noise
		*interrupted noise*	noise with an interval between repeated sounds greater than 1 s
		*impulsive noise*	shocking sounds that appear at once, such as those from dynamite explosions, forging hammer work, etc. noise with a maximum sound pressure level of 120 dB or more, generated at intervals of 1 s or greater maximum acceptance standard for impact sound is 140 dB
		*background noise*	noise in the place when there is no target sound as noise other than the target noise among other types of environmental noise e.g., when focusing on the railway noise at a certain observation point, the traffic noise on a nearby road is considered a background noise, even if the level is higher than the railway noise
		steady noise	almost constant noise with low-noise level changes
		fluctuation noise	noise with an irregular noise level, fluctuating over a wide range
		*intermittent noise*	noise that occurs intermittently, lasting several seconds or longer e.g., train or aircraft noise
		an isolated burst of sound energy	shocking noise that can separate various objects or phenomena, such as driving a pile
		quasisteady impulsive noise	noise produced at short intervals of time with an almost constant level of impact sound, such as a bell or a rock drill

**Table 7:** Expansion of UAM noise standards considering timbre.

Classification of Sounds	Type	Need for UAM Noise Standard Expansion
*timbre*	*continuous noise*	improvement of UAM take-off and landing procedures and methods definition of time periods in which UAM is possible restriction of UAM operation generating high noise enforcing measures to reduce ground noise definition of UAM flight frequency
	*interrupted noise*	
	*impulsive noise*	
	*background noise*	
	*intermittent noise*	

**Table 8:** Requirements to expand noise standards for each UAM flight stage considering timbre.

Requirements to Expand Noise Standards for Each UAM Flight Stage Considering Timbre				
runway use restrictions	improvement of take-off procedures for urban residents	criteria for limiting the operation of aircraft generating high noise restriction on the number of flights restriction on night flights ground noise reduction measures	improvement of landing procedures	restriction on runway use